# Factors Associated with Using Telemedicine in the Primary Care Clinics during the COVID-19 Pandemic in Israel

**DOI:** 10.3390/ijerph192013207

**Published:** 2022-10-14

**Authors:** Orna Reges, Ilan Feldhamer, Yael Wolff Sagy, Gil Lavie

**Affiliations:** 1 Department of Health System Management, Ariel University, Ariel 477625, Israel; 2 Branch of Planning and Strategy, Clalit Health Services, Tel Aviv 6209804, Israel; 3 Ruth and Bruce Rappaport Faculty of Medicine, Technion, Israel Institute of Technology, Haifa 3200003, Israel

**Keywords:** COVID-19 pandemic, telemedicine, primary care visits, health inequality

## Abstract

Background: The COVID-19 pandemic generated an extraordinary need for telemedicine. Objective: To identify the factors and multi-way interactions associated with telemedicine use in primary healthcare during the COVID-19 pandemic. Methods: This population-based study included all members (2,722,773) aged ≥18 years of the largest healthcare organization in Israel who used primary care clinic services between 1 March 2020 and 31 December 2021. Individuals were classified as telemedicine users (≥1 phone/video visits or asynchronous encounters) or non-telemedicine users (only in-person encounter/s). Results: Ethnicity was the most discriminative variable associated with telemedicine use, with 85% and 52% users among Jews and Arabs, respectively. Higher odds for telemedicine utilization were observed among women, residents of urban areas, those confined to home, individuals with high level of technology literacy, residents of the central area (in Jews only), young Jews, and older Arabs. Based on decision tree analysis, the segments of the population with the lowest telemedicine use were characterized by lower primary care needs and comorbidities, as well as low technology literacy. The proportion of telemedicine use in these groups was 56% and 27% in Jews and in Arabs, respectively. Conclusion: A proactive intervention program should be applied among populations who are less likely to use telemedicine in the primary care clinics, including Arabs, Jews who live in the distant periphery, and individuals with low technology literacy.

## 1. Introduction

Advances in communication technology have revolutionized many aspects of business interactions over the last several decades. These advances, together with the worldwide outbreak of coronavirus disease 2019 (COVID-19), have dramatically accelerated the assimilation of telemedicine systems and services within the framework of primary care clinics, as documented over the last 3 years [1,2,3,4,5,6]. Like other countries, during the COVID-19 pandemic, Israel shifted rapidly and to a large extent into virtual health services [7,8]. This included synchronic consultations, that is, encounters that are a real-time interaction between the patient and the physician using a phone or video, as well as asynchronous encounters, which enable the patients to submit any request to the physician and to receive their response at a later time.

Telemedicine enables patients to receive medical treatment without in-person visits to a physician. This can potentially contribute to reducing the inequality in access to medical care [9], but it may also exacerbate disparities in healthcare access for vulnerable populations [10,11]. Indeed, although the provision and coverage of telemedicine were substantially expanded throughout the world [12,13], disparities in using remote clinical services have been documented [4,6,14]. The potential contributing factors include system-related factors, such as lack of telemedicine availability and lack of insurance coverage, in addition to patient-related factors, such as ethnicity, low income, and multimorbidity [11,15,16]. Low technology literacy and limited digital access have been identified as independent factors associated with telemedicine utilization [17,18,19,20] and may be an additional significant barrier to remote clinical services. While the COVID-19 pandemic has accelerated research in the field of telemedicine, more studies are needed to identify the barriers to telemedicine utilization, especially among large multi-ethnic populations. Moreover, beyond the independent association of each potential barrier, the interactions between sociodemographic characteristics and technology literacy should also be considered. Detecting the factors and interactions that have an important role in telemedicine use will enable us to reveal specific subgroups with the lowest probability of using telemedicine.

The objective of this study was to identify, in a large and diverse population, the factors and multi-way interactions associated with telemedicine use in primary healthcare during the COVID-19 pandemic.

## 2. Methods

### 2.1. Data Source

Under the State Health Insurance Law in Israel, each resident is entitled to basic healthcare from one of the four health maintenance organizations (HMO). Clalit Health Services (Clalit) is the largest integrated payer–provider HMO in Israel, which provides care for more than half of the diverse population. The low turnover (about 3% annually) together with the provision of inpatient and outpatient services under one roof enable Clalit to document the vast majority of the healthcare services provided to its members and to maintain an extensive electronic health services data warehouse. This study was approved by the Clalit institutional review board. Since the study was based on historical data from the Clalit electronic data warehouse, the participants’ consent was not required. All patient identities were concealed.

### 2.2. Study Design

This population-based study included all Clalit members who received medical care from a primary care source at least once between 1 March 2020, and 31 December 2021 (the first COVID-19 case in Israel was confirmed on 21 February 2020). All documented encounters, that is, in-person visits, as well as both synchronic and asynchronous telemedicine care encounters were extracted for this study period. The date of the first encounter (in person or telemedicine) was considered as the index date. Baseline characteristics were defined based on the index date.

### 2.3. Study Population

Clalit members, aged ≥18 who received medical care from a primary care source at least once from 1 March 2020, to 31 December 2021, were included in this study. Individuals with no continuous membership at Clalit during this period were excluded from the analysis. Individuals were classified as telemedicine users if they had at least one of the following interactions with the primary care clinic: (1) scheduled phone encounter with their primary care physician, (2) scheduled video encounter with their primary care physician, or (3) utilization of asynchronous telemedicine care service. Individuals were classified as non-telemedicine users if they had only in-person encounter/s during the study period.

### 2.4. Study Variables

The sociodemographic variables assessed at the index date included sex, age (years), socio-economic status (SES), birthplace (Israel, Other), number of children, peripherality index (central, proximate periphery, distant periphery), and residence in an urban area (yes/no). SES was defined based on the Israeli Central Bureau of Statistics’ small statistical areas (SSAs) [21]—20 socio-economic categories defined based on household income, housing conditions, demography, and education, as measured in the 2008 Israeli census. For this study, updated categories from the POINTSC Location Intelligence Company were used [22]. These POINTS updated categories were based on socio-economic characteristics measured in 2019 and were expressed on 10 levels of SES ranging from 1 (lowest) to 10 (highest). The final SES variable included in this study was defined as four categories (1–3, 4–5, 6–7, and 8–10). Missing data on SES were imputed based on the primary care clinic level, according to the clinic location. The peripherality index was defined based on the Israeli CBS Peripherality Index of Local Authorities, which categorized local authorities in Israel based on their proximity to the central economic activity in Israel. The index takes into account the potential accessibility index (that is, a combination of the proximity of the local authority to all the local authorities in the country with the size of the population in the local authority) and the proximity to the Tel Aviv District [23]. Health status was measured by using two variables, including the Johns Hopkins Adjusted Clinical Group (ACG) Comorbidity score [24] and home confinement for medical reasons (yes/no). As an estimate of technological literacy, the existence of an e-mail address available for Clalit Health Service was examined (y/n).

### 2.5. Statistical Analysis

Statistical analyses were carried out using Python 3.7.6 software.

The baseline characteristics of the study population were described by using proportions for categorical variables and means with standard deviation (SD) for continuous variables.

The univariable association of each of the baseline characteristics with telemedicine use in primary healthcare was assessed by using chi-square tests and *t*-tests for categorical and continuous variables, respectively.

In order to explore the independent associations of sociodemographic characteristics, health status, and technological literacy with telemedicine use in primary healthcare, multivariable logistic models, stratified by ethnic groups, were applied. Correlation coefficients were calculated to evaluate the presence of multicollinearity between the baseline characteristics.

In order to expose the best discriminative characteristics and multi-way interactions associated with telemedicine use in primary healthcare during the COVID-19 pandemic, a decision tree analysis was applied. Sociodemographic characteristics, health status, and technological literacy assessed at index date were included in the analysis. The dataset was divided into training (70%) and testing (30%) samples. The classification and regression tree (CART) algorithm, which produces binary splits and forms a relatively small tree, was selected as a growing method. The Gini index was used for feature selection. The cost complexity pruning approach was used to avoid overfitting in building the decision tree.

## 3. Results

From 1 March 2020, to 31 December 2021, there were 2,986,140 Clalit members aged ≥ 18 who received medical care from a primary care source at least once during the study period. Of these, 263,367 individuals with no continuous membership at Clalit during this period were excluded from the analysis. The final study population included 2,722,773 members: 1,990,049 Jews and 732,724 Arabs. The main characteristics of the study population by population group are summarized in Table 1. Arab participants, compared to Jewish participants, were of younger age (mean age 39.7 vs. 49.4 years, respectively) and, on average, had more children (1.11 vs. 0.78, respectively), lower SES level (3.27 vs. 6.09, respectively), and lower comorbidity score (2.87 vs. 3.15, respectively). The proportion of individuals who live in the distant periphery was higher among Arab participants (34.3% vs.14.2%). Jewish participants were more likely to have provided Clalit with their e-mail address (77.8% vs. 37.0%).

Of the total study population, 2,073,584 (76%) used synchronic or asynchronous telemedicine at least once during the study period. Significant difference (*p* < 0.001) in telemedicine use was observed between the Jewish and the Arab population, with 85% and 52% of users, respectively (Table 1).

Table S1 and Table 2 describe the association of personal factors with telemedicine use in primary healthcare during the COVID-19 pandemic, stratified by ethnic groups.

Among both Jewish and Arab participants, a greater use was observed among women, residents of urban areas, those confined to home, and those who have provided their personal e-mail address to Clalit. The association of all these factors was stronger among Jewish compared with Arab participants. Multivariable logistic models (Table 2) revealed differences between Jewish and Arab participants in factors associated with telemedicine use in primary healthcare during the COVID-19 pandemic. Among the Jewish population, compared with middle-aged participants (45–64 yrs.), telemedicine use was higher among young individuals aged 18–24 yrs. (OR, 95%CI: 1.14, 1.14–1.14) and lower among old adults aged ≥ 75 yrs. (OR, 95%CI: 0.97, 0.97–0.97). The opposite association was demonstrated among the Arab population, with lower use among younger individuals (OR, 95%CI: 0.93, 0.93–0.93) and higher use among older adults (OR, 95%CI: 1.17, 1.17–1.17). While among Jews, compared with those living in the distant periphery, the odds for telemedicine use were higher among those living in the proximate periphery (OR, 95%CI: 1.56, 1.56–1.56) and highest among those living in the central area (OR, 95%CI: 1.72, 1.71–1.72), peripherality was not revealed as an important variable associated with telemedicine use among the Arab population (*p* < 0.05 for all interaction terms).

**Figure 1:**
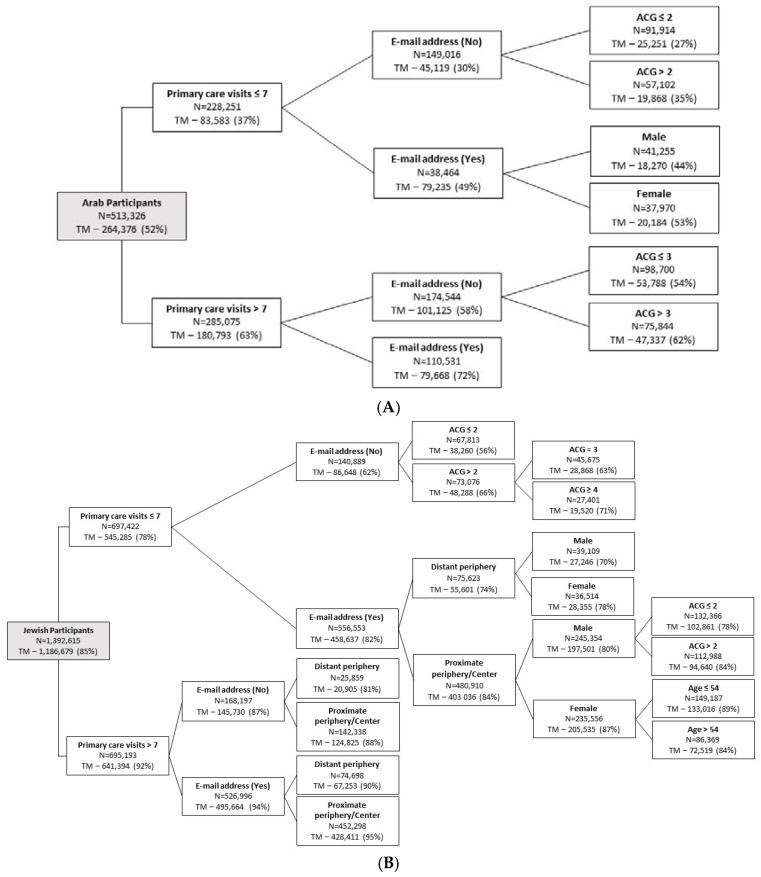
Classification tree analysis for utilization of telemedicine during the COVID-19 pandemic. (**A**) Arab participants; (**B**) Jewish participants.

Using a decision tree analysis (Figure 1), the most important variables associated with using telemedicine during the COVID-19 pandemic were ethnicity, number of interactions with the primary care clinics (telemedicine or in person), and technology literacy, measured by the existence of e-mail address available to the Clalit Health Service. Within the Arab population, compared to 52% telemedicine users in total, only 27% of those with lower primary care needs (<7 interactions during the study period), with no e-mail address available for the Clalit Health Service, and with lower level of comorbidity (ACG score ≤ 2) used any telemedicine care service during the study period. On the other hand, the proportion of telemedicine users increased to 62% among Arabs who had greater primary care needs (>7 interactions during the study period), higher level of comorbidity (ACG score > 3), and an e-mail address available to the Clalit Health Service. These three variables, i.e., the number of patient–physician interactions, technology literacy, and comorbidity level, were also found to be the most important variables among the Jewish population. Specifically, Jews who needed primary care clinic services ≤ 7 times during the study period, had no e-mail address available to the Clalit Health Service, and had a lower level of comorbidity (ACG score ≤ 2) were revealed as a group with the lowest probability of using telemedicine services (56%). On the other hand, the utilization of telemedicine increased from 85% in total to 95% among Jews who had greater primary care needs (>7 interactions during the study period), high level of technology literacy, and lived in the proximal periphery or in the center.

## 4. Discussion

**Figure 2:**
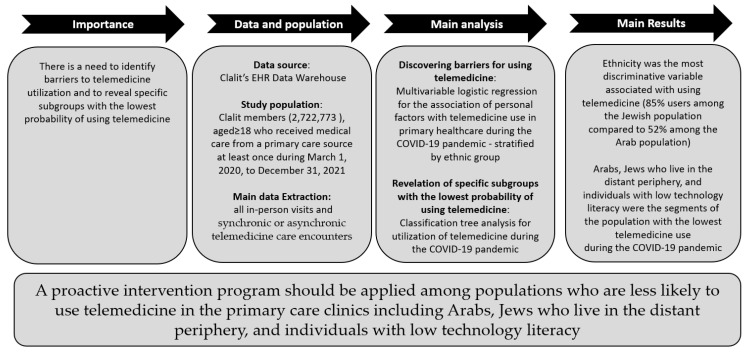
General research framework.

In this study, of the 2,722,773 Clalit members aged ≥ 18 who needed primary care clinic services between 1 March 2020, and 31 December 2021, 76% used synchronic or asynchronous telemedicine at least once during the study period. Ethnicity was the most discriminative variable associated with using telemedicine during the COVID-19 pandemic, with 85% of users among the Jewish population compared to only 52% among the Arab population. Among both population groups, a greater use was observed among women, residents of urban areas, those confined to home, and those who have provided a personal e-mail address to Clalit. Peripherality was revealed as an important variable only among the Jewish population with increased odds for telemedicine use among those living in the central area. The odds for using telemedicine were higher among young Jews and old Arabs. Among both Jews and Arabs, those who had lower primary care needs, low technology literacy, and low level of comorbidity were the segment of the population with the lowest telemedicine use, with 56% and 27% of users, respectively. A general framework of the study is presented in Figure 2.

Similar to previous studies [15,16], this study demonstrated significant ethnic differences in the adoption of telemedicine during the COVID-19 pandemic, with lower use among ethnic minorities. A recently published study [15] demonstrated lower telemedicine use among African Americans, Asians, and American Indians compared with Whites (OR 95% CI: = 0.65, 0.61, 0.69; 0.58, 0.52, 0.65; and 0.82 0.70, 0.98, respectively) during the COVID-19 pandemic. Lower use of technology for managing health was also observed among older racial and ethnic minorities before the COVID-19 pandemic [25]. These findings support the implementation of proactive intervention programs to remove barriers to the use of telemedicine among ethnic minority groups. In addition, the origin of the major ethnic disparity in the use of telemedicine should be examined in future studies. It is possible that personal and ethnic characteristics, such as emotional status, preferences, perceptions, and cultural determinants, may play a role in using telemedicine and may explain some of the differences in the use of telemedicine between the ethnic groups. Previous studies demonstrated differences between Jews and Arabs in these characteristics and their association with health status and utilization of health services [26,27]. In contrast, a study by Campos-Castillo et al. [28] on racial and ethnic differences in telehealth use during the COVID-19 pandemic demonstrated higher synchronous and asynchronous telehealth use among Black compared to White participants. That study, however, included only internet users and cannot be generalized to individuals without internet access, including those with low technology literacy, low income, and living in rural areas.

Among both Jews and Arabs, low technology literacy was revealed as a significant barrier to telemedicine use. This finding confirmed the concern raised by primary care clinicians regarding the disparities in access to primary care for individuals with limited technology literacy or access [10]. As Nouri et al. [10] emphasized, it is particularly important to address this barrier in the primary care where continuous communication is a crucial factor for optimal treatment, especially among patients with comorbidities. In this study, about one-third of the population had a low level of technology literacy, which was manifested by the absence of an e-mail address available to the Clalit Health Service, with significant differences between the Jewish (22.2%) and the Arab (66.0%) populations.

While telemedicine has the potential to make healthcare services more accessible in peripheral and rural areas, lower odds for telemedicine use were observed among non-urban residents and among Jews living in the periphery. A study by Dopelt et al. [29] found that individuals from a peripheral clinic in Israel had sufficient knowledge about how to utilize the internet for health purposes but did not feel confident using it to make health decisions and preferred personal interaction after the epidemic’s end. More studies are needed to identify the additional obstacles to telemedicine use among those living in the periphery.

An interesting finding in this study was the higher use among older Arabs. This finding is inconsistent with previous findings that have shown higher use among younger patients [11]. Further investigation on the association of age with telemedicine use among the Arab population is required.

The most significant advantage of this study was its ability to capture all health services utilized during the COVID-19 pandemic by 2,722,773 Clalit members and to distinguish between telemedicine users and non-users. This is based on a detailed documentation of the majority of the healthcare services provided to Clalit members and by maintaining an extensive electronic health services data warehouse. In addition, in many places, coverage and insurance reimbursement for telemedicine use play a critical role in the decision to use this service. In Israel, however, under the State Health Insurance Law, each resident is entitled to basic healthcare, which includes free-of-charge access to primary care clinics. This option enabled the study of barriers to telemedicine use beyond the payment barrier.

This study has several limitations. First, it was impossible to identify non-telemedicine-eligible visits, i.e., visits in which physical examination was required. Second, the existence of an e-mail address and making it available to Clalit was used as a proxy for technological literacy. Although this may indicate some technological proficiency, and perhaps the ability to use digital devices and interfaces, and although it is considered a digital skill [30], it does not necessarily indicate technological literacy. Third, the study is limited to primary care clinics in Israel, and the results may not be generalizable to other countries.

The results of this study empower future research to examine the origin of the difference in the use of telemedicine between Jews and Arabs. Information regarding the impacts of using telemedicine on the quality of healthcare services and patient satisfaction may provide a more complete overview of the impact of assimilation of telemedicine systems and services within the framework of primary care clinics.

## 5. Conclusions

The COVID-19 pandemic generated an extraordinary need for telemedicine, which potentially contributes to reducing the inequality in access to medical care. However, other barriers may exacerbate the disparities. This study identified vulnerable populations who were less likely to use telemedicine in the primary care clinics during the COVID-19 pandemic. These populations included Arabs, Jews who lived in the distant periphery, and individuals with low technology literacy. Proactive intervention programs should be applied among these groups.

## Figures and Tables

**Table 1:** Main characteristics of the study population.

		Total	Jewish	Arabs
		*n* = 2,722,773	*n* = 1,990,049	*n* = 732,724
Sex				
Male	*n* (%)	1,276,981 (46.9%)	922,694 (46.4%)	354,287 (48.4%)
Female	*n* (%)	1,445,792 (53.1%)	1,067,355 (53.6%)	378,437 (51.6%)
Age				
Years	Mean (SD)	46.8 (18.4)	49.4 (18.5)	39.7 (16.3)
18–24	*n* (%)	294,793 (10.8%)	138,633 (7.0%)	156,160 (21.3%)
25–44	*n* (%)	1,103,065 (40.5%)	787,219 (39.6%)	315,846 (43.1%)
45–64	*n* (%)	754,296 (27.7%)	558,906 (28.1%)	195,390 (26.7%)
65–74	*n* (%)	343,734 (12.6%)	301,296 (15.1%)	42,438 (5.8%)
≥75	*n* (%)	226,885 (8.3%)	203,995 (10.3%)	22,890 (3.1%)
Socio-economic status				
Level	Mean (SD)	5.34 (2.08)	6.09 (1.79)	3.27 (1.26)
1–3	*n* (%)	689,683 (25.3%)	191,052 (9.6%)	498,631 (68.1%)
4–5	*n* (%)	731,923 (26.9%)	543,824 (27.3%)	188,099 (25.7%)
6–7	*n* (%)	807,750 (29.7%)	768,017 (38.6%)	39,733 (5.4%)
8–10	*n* (%)	493,417 (18.1%)	487,156 (24.5%)	6,261 (0.9%)
Birthplace				
Israel	*n* (%)	2,053,293 (75.4%)	1,335,968 (67.1%)	717,325 (97.9%)
Other	*n* (%)	669,480 (24.6%)	654,081 (32.9%)	15,399 (2.1%)
Children				
Number	Mean (SD)	0.86 (1.43)	0.78 (1.36)	1.11 (1.56)
0	*n* (%)	1,757,101 (64.5%)	1,345,735 (67.6%)	411,366 (56.1%)
1–2	*n* (%)	568,934 (20.9%)	387,863 (19.5%)	181,071 (24.7%)
3–4	*n* (%)	327,503 (12.0%)	214,355 (10.8%)	113,148 (15.4%)
≥5	*n* (%)	69,235 (2.5%)	42,096 (2.1%)	27,139 (3.7%)
Peripherality index				
Distant periphery (1–4)	*n* (%)	533,160 (19.6%)	281,677 (14.2%)	251,483 (34.3%)
Proximate periphery (5–7)	*n* (%)	1,239,005 (45.5%)	898,034 (45.1%)	340,971 (46.5%)
Central (8–10)	*n* (%)	950,608 (34.9%)	810,338 (40.7%)	140,270 (19.1%)
Residence in an urban area				
No	*n* (%)	809,988 (29.7%)	428,781 (21.5%)	381,207 (52.0%)
Yes	*n* (%)	1,912,785 (70.3%)	1,561,268 (78.5%)	351,517 (48.0%)
ACG Comorbidity Score				
Level	Mean (SD)	3.07 (1.11)	3.15 (1.11)	2.87 (1.09)
1–2	*n* (%)	932,464 (34.2%)	632,895 (31.8%)	299,569 (40.9%)
3	*n* (%)	855,588 (31.4%)	622,940 (31.3%)	232,648 (31.8%)
4	*n* (%)	650,732 (23.9%)	502,875 (25.3%)	147,857 (20.2%)
5–6	*n* (%)	283,989 (10.4%)	231,339 (11.6%)	52,650 (7.2%)
Home confinement				
No	*n* (%)	2,125,820 (97.6%)	1,606,635 (97.4%)	519,185 (98.1%)
Yes	*n* (%)	52,386 (2.4%)	42,368 (2.6%)	10,018 (1.9%)
E-mail address				
Yes	*n* (%)	1,819,024 (66.8%)	1,548,243 (77.8%)	270,781 (37.0%)
No	*n* (%)	903,749 (33.2%)	441,806 (22.2%)	461,943 (63.0%)
Primary care visits				
Number of visits	Mean (SD)	10.67 (10.15)	10.17 (9.46)	12.05 (11.72)
Telemedicine utilization				
≥1 Telemedicine care service	*n* (%)	2,073,584 (76%)	1,696,225 (85%)	377,359 (52%)

**Table 2:** Multivariable logistic regression for the association of personal factors with telemedicine use in primary healthcare during the COVID-19 pandemic stratified by ethnic group.

	Jewish *n* = 1,990,049	Arabs *n* = 732,724
Predictor	OR (95% CI)	OR (95% CI)
Sex		
Male (Ref. group)	1.00	1.00
Female	1.39 (1.39–1.39)	1.28 (1.28–1.28)
Age (years)		
18–24	1.14 (1.14–1.14)	0.93 (0.93–0.93)
25–44	1.28 (1.28–1.28)	1.06 (1.06–1.06)
45–64 (Ref. group)	1.00	1.00
65–74	0.89 (0.89–0.89)	0.94 (0.94–0.94)
≥75	0.97 (0.97–0.97)	1.17 (1.17–1.17)
Socio-economic status		
1–3 (Ref. group)	1.00	1.00
4–5	1.10 (1.10–1.10)	1.03 (1.03–1.03)
6–7	1.23 (1.23–1.23)	1.62 (1.62–1.62)
8–10	1.27 (1.27–1.27)	1.30 (1.30–1.30)
Birthplace		
Israel	1.31 (1.31–1.31)	N/A
Other (Ref. group)	1.00	
Children		
0 (Ref. group)	1.00	1.00
1–2	1.09 (1.09–1.09)	1.05 (1.05–1.05)
3–4	1.07 (1.07–1.07)	1.05 (1.05–1.05)
≥5	1.12 (1.12–1.12)	1.18 (1.18–1.18)
Peripherality index		
Distant periphery (1–4) (Ref. group)	1.00	1.00
Proximate periphery (5–7)	1.56 (1.56–1.56)	1.08 (1.08–1.08)
Central (8–10)	1.72 (1.72–1.72)	0.98 (0.98–0.98)
Residence in an urban area		
No (Ref. group)	1.00	1.00
Yes	1.40 (1.40–1.40)	1.19 (1.19–1.19)
ACG Comorbidity Score		
1–2 (Ref. group)	1.00	1.00
3	1.28 (1.28–1.28)	1.16 (1.16–1.16)
4	1.58 (1.58–1.58)	1.27 (1.27–1.27)
5–6	1.64 (1.64–1.64)	1.28 (1.28–1.28)
Home confinement		
No (Ref. group)	1.00	1.00
Yes	1.96 (1.96–1.96)	1.42 (1.42–1.42)
E-mail address		
No (Ref. group)	1.00	1.00
Yes	2.49 (2.49–2.49)	2.01 (2.01–2.01)
Primary care visits		
Number of visits	1.11 (1.11–1.11)	1.06 (1.06–1.06)

No multicollinearity between the predictors was observed (Pearson/Cramer correlation coefficients < 0.63).

## Data Availability

The data are not publicly available due to privacy restrictions.

## Supplementary Materials

The following supporting information can be downloaded at: https://www.mdpi.com/article/10.3390/ijerph192013207/s1, Table S1: Univariable analysis for the association of personal factors with telemedicine use in primary healthcare during the COVID-19 pandemic—stratified by ethnic group.
