# Supplementary material for: Factors Associated with Using Telemedicine in the Primary Care Clinics during the COVID-19 Pandemic in Israel
Source: Int J Environ Res Public Health. 2022 Oct 14;19(20):13207. doi: 10.3390/ijerph192013207 (PMC9603207; doi:10.3390/ijerph192013207)
Supplement: Supplementary file 1 [file ijerph-19-13207-s001.zip › ijerph-1910603-supplementary.pdf]

Table S1. Univariable analysis for the association of personal factors with telemedicine use in primary healthcare during the COVID-19 pandemic - stratified by ethnic group.

		Total		Jewish		Arabs	
	Tele-Use	**Yes**	No	**Yes**	No	**Yes**	No
**Sex**							
Male	%	72%	28%	81%	19%	46%	54%
Female	%	80%	20%	89%	11%	57%	43%
**Age**							
Years	Mean (SD)	48.0 (18.5)	42.8 (17.5)	49.6 (18.6)	48.5 (17.8)	41.2 (16.6)	38.1 (15.8)
18-24	n (%)	61%	39%	83%	17%	43%	57%
25-44	n (%)	76%	24%	86%	14%	52%	48%
45-64	n (%)	76%	24%	84%	16%	54%	46%
65-74	n (%)	82%	18%	85%	15%	59%	41%
≥75	n (%)	86%	14%	89%	11%	65%	35%
**Socioeconomic status**							
Level	Mean (SD)	5.6 (2.0)	4.4 (2.0)	6.1 (1.8)	5.9 (1.8)	3.3 (1.3)	3.2 (1.2)
1-3	n (%)	59%	41%	81%	19%	50%	50%
4-5	n (%)	76%	24%	84%	16%	53%	47%
6-7	n (%)	85%	15%	86%	14%	64%	36%
8-10	n (%)	87%	13%	87%	13%	59%	41%
**Birthplace**							
Israel	n (%)	74%	26%	86%	14%	51%	49%
Other	n (%)	84%	16%	84%	16%	65%	35%
**Children**							

Number	Mean (SD)	0.8 (1.4)	0.9 (1.5)	0.8 (1.4)	0.7 (1.4)	1.1 (1.6)	1.1 (1.6)
0	n (%)	77%	23%	85%	15%	50%	50%
1-2	n (%)	76%	24%	87%	13%	54%	46%
3-4	n (%)	74%	26%	86%	14%	53%	47%
≥5	n (%)	72%	28%	84%	16%	52%	48%
**Peripherally index**							
Distant periphery (1-4)	n (%)	65%	35%	79%	21%	50%	50%
proximate periphery (5-7)	n (%)	76%	24%	85%	15%	52%	48%
Central (8-10)	n (%)	82%	18%	87%	13%	53%	47%
**Residence in an urban area**							
No	n (%)	67%	33%	82%	18%	50%	50%
Yes	n (%)	80%	20%	86%	14%	53%	47%
**ACG Comorbidity Score**							
Level	Mean (SD)	3.2 (1.1)	2.7 (1.1)	3.2 (1.1)	2.7 (1.1)	3.1 (1.1)	2.7 (1.0)
1-2	n (%)	67%	33%	79%	21%	42%	58%
3	n (%)	76%	24%	85%	15%	53%	47%
4	n (%)	84%	16%	90%	10%	63%	37%
5-6	n (%)	88%	12%	92%	8%	67%	33%
**Home confinement**							
No	n (%)	78%	22%	86%	14%	53%	47%
Yes	n (%)	88%	12%	93%	7%	68%	32%
**E-mail address**							
Yes	n (%)	84%	16%	88%	12%	62%	38%
No	n (%)	60%	40%	75%	25%	45%	55%
